# Reconstruction of Simplified Microbial Consortia to Modulate Sensory Quality of Kombucha Tea

**DOI:** 10.3390/foods11193045

**Published:** 2022-09-30

**Authors:** Nicola Ferremi Leali, Renato L. Binati, Francesco Martelli, Veronica Gatto, Giovanni Luzzini, Andrea Salini, Davide Slaghenaufi, Salvatore Fusco, Maurizio Ugliano, Sandra Torriani, Elisa Salvetti

**Affiliations:** Department of Biotechnology, University of Verona, 37134 Verona, Italy

**Keywords:** kombucha tea, SCOBY, simplified microbial consortium, *Novacetimonas hansenii*, *Brettanomyces bruxellensis*, *Zygosaccharomyces parabailii*, bacterial cellulose, fermentation metabolites, volatile organic compounds, sorting task

## Abstract

Kombucha is a fermented tea with a long history of production and consumption. It has been gaining popularity thanks to its refreshing taste and assumed beneficial properties. The microbial community responsible for tea fermentation—acetic acid bacteria (AAB), yeasts, and lactic acid bacteria (LAB)—is mainly found embedded in an extracellular cellulosic matrix located at the liquid–air interphase. To optimize the production process and investigate the contribution of individual strains, a collection of 26 unique strains was established from an artisanal-scale kombucha production; it included 13 AAB, 12 yeasts, and one LAB. Among these, distinctive strains, namely *Novacetimonas hansenii* T7SS-4G1, *Brettanomyces bruxellensis* T7SB-5W6, and *Zygosaccharomyces parabailii* T7SS-4W1, were used in mono- and co-culture fermentations. The monocultures highlighted important species-specific differences in the metabolism of sugars and organic acids, while binary co-cultures demonstrated the roles played by bacteria and yeasts in the production of cellulose and typical volatile acidity. Aroma complexity and sensory perception were comparable between reconstructed (with the three strains) and native microbial consortia. This study provided a broad picture of the strains’ metabolic signatures, facilitating the standardization of kombucha production in order to obtain a product with desired characteristics by modulating strains presence or abundance.

## 1. Introduction

Kombucha is a traditional beverage of Asian origin, made by fermenting sugared green or black tea with a symbiotic culture of bacteria and yeasts within a cellulosic matrix. It has a long history of production and consumption that traces back to 220 BCE during the Tsin Dynasty in China, when it was considered a beverage with detoxifying and energizing properties [1]. In recent years, kombucha consumption has grown in North America and Europe due to its refreshing taste and associated beneficial health properties which include, among others, antioxidant, antitumoral, anti-inflammatory, and hepatoprotective effects, digestion improvement, and microbial infection prevention [1]. Even though these have yet to be fully proven in human clinical trials, these claims have contributed to the high popularity of kombucha [2].

The microbial consortium responsible for tea fermentation is commonly named SCOBY (Symbiotic Culture of Bacteria and Yeasts) and is composed by acetic acid bacteria (AAB), yeasts, and lactic acid bacteria (LAB) [3]. These microorganisms are embedded in an extracellular cellulosic matrix located at the liquid–air interphase. Several studies of kombucha fermentations have shown different microbial compositions of the SCOBY, which can vary depending on substrate, origin, climate, geographic location, and production conditions [2,4]. The most abundant yeasts present in the symbiotic culture belonged to the genera *Brettanomyces/Dekkera* and *Zygosaccharomyces*, while some reports also found *Saccharomyces*, *Schizosaccharomyces*, and *Hanseniaspora*, among others. Regarding AAB, *Komagataeibacter*, *Novacetimonas* (a recently described genus including a group of former *Komagataeibacter* spp. [5]), *Acetobacter*, and *Gluconobacter* were prevalent, while among LAB, although not always present, *Oenococcus* and *Liquorilactobacillus* (former *Lactobacillus* spp. [6]) were the dominant genera [3,7].

The scientific exploration of this fermented beverage is quite difficult due to the huge diversity and abundance of microorganisms involved in its production, as well as the metabolic interactions between them. Nevertheless, some members of the consortium have a well-defined role in cooperative metabolism between the species, contributing to the chemical composition of kombucha. In fact, at the beginning of the fermentation process, the yeast community hydrolyzes sucrose into glucose and fructose by periplasmatic invertase and produces ethanol; this compound is metabolized by AAB to acetic acid, conferring the characteristic acidic aroma and flavor of vinegar to kombucha [8]. Glucose and fructose are also used by AAB to produce cellulose, leading to the formation of a cellulosic matrix [8]. Particularly, some species, belonging to the genera *Komagataeibacter* and *Novacetimonas*, such as *Komagataeibacter xylinus*, *Novacetimonas hansenii*, and *Novacetimonas maltaceti*, were found to be very efficient producers of this biopolymer [5,9]. This matrix is an adhesion surface and protects the embedded cells from unfavorable environmental factors, such as ultraviolet radiation or heat [8]. Furthermore, it exposes the AAB to the aerobic environment, which is essential for their growth and metabolism. The role of LAB has been mainly linked to their ability to produce lactic and gluconic acids that enhance the antimicrobial and antioxidant properties of kombucha—and could also impact the overall sensory perception [10]. These bacteria were reported to comprise up to 30% of the bacterial population of kombucha cultures [2], even though several surveys have not detected their presence at all [3,11,12].

The chemical composition of kombucha has been largely described in literature [13,14], revealing that some compounds, such as organic acids, vitamins, polyphenols, and amino acids, are present at high concentrations [15]. However, ample variations in composition and metabolite concentration have been observed, as they depend on several factors, such as type of tea, fermentation time, and microorganisms of the inoculum [16]. Indeed, several laboratory fermentations of kombucha using different SCOBY consortia and tea brews led to a broad range of beverages that differed in terms of their chemical composition, including the relative amount of ethanol and acetic acid [7,17]. Some studies also analyzed the volatile organic compounds (VOCs) of kombucha, which, in addition to confirming variability between different batches, revealed that acids and esters were the most important contributors to overall organoleptic properties, as they were associated with the common descriptors of the product, e.g., acidic, refreshing, and possessing a fruity aroma [9,10]. Quality attributes related to VOCs have defined taste panel protocols for sensory analysis, selecting appropriate descriptors (i.e., sweetness, sourness, bitterness) [18]. Kombucha tea taste is generally described by evaluators as pleasantly acidic and harmonic, with a fresh, sour, fruity, and vinegar-like flavor [19,20].

High reproducibility and control of the fermentation process help to ensure a better quality of the final product, but the complex nature of the SCOBY traditionally used to start new fermentations makes it difficult to standardize Kombucha production. Indeed, the production of batches by successive propagation could lead to the evolution of the complex microbial consortium in terms of composition, microbial dynamics, or both [21]. The fluctuation of biological components could lead to unstable qualities of kombucha fermentation kinetics and chemical composition and, consequently, organoleptic properties [22]. To overcome this, it is necessary to understand the role of each microbial component of SCOBY, depicting its contribution to the final organoleptic characteristics of the beverage, and selecting the most significant species and strains to simplify kombucha consortia. Indeed, a limited number of strains with desirable characteristics, such as *B. bruxellensis* and *K. rhaeticus* strains, can produce a kombucha-like fermentation without other taxa [10,23].

In this context, the present study aimed to investigate the microbial structure of an artisanal kombucha, with the ultimate goal of establishing a collection of taxonomically well-defined strains to be used for an ad hoc consortium development. Adopting a microbiota deconstruction–reconstruction approach in a controlled laboratory environment, the dominant and peculiar bacteria and yeast strains were selected, and their contribution to the aromatic profile and organic acid production was explored. The derived tailor-made microbial community could be used to standardize the production process and the quality of the final product.

## 2. Materials and Methods

### 2.1. Analyzed Samples, Microbial Enumeration and Isolation

Samples were collected during the manufacture of kombucha at an artisanal producer located in Verona, Italy. The fermented beverage was prepared by infusing a 0.3% *w/v* blend of green and black tea (80% and 20%, respectively) with 5% *w*/*v* brown sugar, and a kombucha culture from a previous batch (20% *v*/*v* liquid phase and 0.28% *w*/*v* SCOBY), which was used to inoculate microorganisms and decrease the pH below 4.6. The fermentation was carried out in a dedicated room with controlled temperature (28 ± 1 °C), to ensure better growth conditions and to limit the occurrence of possible contaminations. A total of 11 samples were collected in November 2020 from five tanks; three of them, i.e., T2 (21 days of fermentation), T3 and T4 (14 days), were used for production of kombucha and the other two (T1, T5) for SCOBY maintenance and propagation. SCOBY was maintained in a smaller tank (T1), submerged in tea, while for propagation, another tank (T5) with an increased liquid/air interface area was used. Samples were distinguished in SCOBY (seven samples) and liquid phase (four samples). All samples were transferred to the laboratory in refrigerated conditions and stored at 4 °C.

For microbiological analysis, SCOBY samples (5 g) were first resuspended in 5 mL physiological solution (0.9% *w*/*v* NaCl) and then disrupted with an ULTRA-TURRAX disperser (IKA-Werke, Staufen, Germany). Decimal dilutions in physiological solution of homogenized SCOBY and liquid phase samples were then plated on Wallerstein Lab (WL) agar medium (ThermoFisher Scientific, Waltham, MA, USA) supplemented with chloramphenicol (100 mg/L) for yeasts, MRS agar (Oxoid, Basingstoke, England, UK) for LAB, and GYC (50 g/L glucose, 10 g/L yeast extract, 30 g/L CaCO_3_, 25 g/L agar) supplemented with 100 mg/L cycloheximide and 100 mg/L nystatin for AAB. All media were incubated at 28 °C for up to 5 days. MRS plates were incubated anaerobically with Anaerocult^TM^ A (Merck, Darmstadt, Germany). After counting, representative colonies were picked up and purified by successive streaking on their respective isolation media. Putative yeasts, AAB, and LAB isolates were routinely grown at 28 °C for three days in YPD (10 g/L yeast extract, 20 g/L bacteriological peptone, 20 g/L glucose), GY (50 g/L glucose, 10 g/L yeast extract), and MRS broth, respectively. Isolates were stored at −80 °C in 25% *v*/*v* glycerol (Thermo Scientific, Monza, Italy) solution. Unless otherwise specified, reagents were purchased from Sigma-Aldrich (Milan, Italy).

### 2.2. DNA Extraction and Taxonomic Identification

Total genomic DNA was obtained from a 2-mL aliquot of three-days-grown AAB, LAB, and yeast cultures. Briefly, cells were harvested by centrifugation (14,000 rpm for 10 min), washed with distilled water, and then resuspended in 10 mg/µL lysozyme solution (Sigma-Aldrich), for bacterial isolates, or 10 U/µL lyticase solution (Sigma-Aldrich), for yeasts. After incubation (37 °C, 1 h), DNA isolation followed the protocol of Wizard Genomic DNA Purification Kit (Promega, Milan, Italy). DNA yield and purity were determined with a NanoDrop ND1000 UV–Vis Spectrophotometer (Thermo Scientific, Waltham, MA, USA), and DNA samples were stored at −20 °C for downstream analysis.

Rep-PCR with primer (GTG)_5_ was employed according to the protocol of [24], modified by [25,26]. PCR amplification was conducted in a Thermal Cycler 2720 (Applied Biosystems, Foster City, CA, USA) with the following program for bacterial DNA: 5 min of denaturation at 94 °C, 30 cycles of 1 min at 95 °C, 1 min at 40 °C, and 8 min at 72 °C, then 16 min at 72 °C for the final elongation. As for yeast DNA, the program was modified as follows: 5 min at 94 °C, 35 cycles of 50 s at 95 °C, 50 s at 40 °C, and 90 s at 72 °C, then 5 min at 72 °C. The products were run on a 1.5% *w*/*v* agarose gel in 1× TAE buffer (40 mM Tris, 20 mM acetic acid, and 0.4 mM EDTA) stained with Atlas Clearsight (Bioatlas, Tartu, Estonia) at 110 V for 2 h. Each gel was loaded with the molecular ladder O’Gene Ruler DNA (Thermo Scientific) to normalize the runs.

Visualization and image capturing were made under UV light with the UVITEC Gel Documentation System (Cleaver Scientific, Rugby, England). Fingerprinting data were then analyzed using Bionumerics v.7.6. (Applied Maths, Sint-Martens-Latem, Belgium), with the Pearson’s correlation coefficient and the unweighted pair group method with arithmetical average (UPGMA) clustering method.

Identification of representative strains of different Rep-PCR clusters were done by 16S rRNA gene and 26S rRNA gene sequencing for LAB and yeasts, respectively. Identification of AAB were performed through *dnaK* gene sequencing, due to the high similarity between 16S rRNA gene of different AAB species. The 16S rRNA gene was amplified using the primers E8F and E1541R, according to [27], while the D1/D2 domain of the 26S rRNA gene was amplified using primers NL-1 and NL-4, following the protocol of [28].

The gene *dnaK* was amplified by the newly designed primers DNAKF (fwd-TGAAGTGCTGCGTATCATCAACGA) and DNAKR (rev-ATTTCACGTTCGCCCTGATA) with the following program: 5 min of denaturation at 95 °C, 30 cycles of 30 s at 95 °C, 30 s at 60 °C, and 1 min at 72 °C, then 10 min at 72 °C for the final elongation, using the following reagent concentrations: DreamTaq Green Buffer (Thermo Scientific) 1X, dNTPs 250 μM, primer DNAKF 1 μM, primer DNKAR 1 μM, Dream Taq (Thermo Scientific) 0.025 U/μL, DNA 50 ng/μL. The PCR products were purified and sent for sequencing at Eurofins Genomics (Ebersberg, Germany). Yeasts and AAB sequencing data were searched against the GenBank database using the BLAST alignment tool (http://blast.ncbi.nlm.nih.gov/, accessed on 4 July 2022), while 16S rRNA gene sequencing data were searched against bacterial type strains 16S rRNA gene sequences in the EzBioCloud database (https://www.ezbiocloud.net/, accessed on 4 July 2022). The sequencing data generated in this study were all deposited in the NCBI database within the projects n. BankIt2616791 for *dnaK* gene sequences of AAB, SUB11972523 for 16S rRNA gene sequences of LAB, and SUB11972681 for 26S rRNA gene sequences of yeasts.

### 2.3. Fermentation Trials

Lab-scale fermentation trials were carried out with mono- and co-cultures of distinctive strains: *B. bruxellensis* T7SB-5W6, *N. hansenii* T7SS-4G1, and *Z. parabailii* T7SS-4W1. They were compared with the native microbial consortium used for kombucha manufacture by the artisanal producer. Sugared tea (2.5% *w*/*v* fructose and 2.5% *w*/*v* glucose) was prepared by infusing 0.3% *w*/*v* tea leaves (80% green and 20% black) in water at 85 °C for 5 min and acidifying with acetic acid (Sigma-Aldrich) to bring the pH below 4.6. To prepare the inoculum, cells were cultivated in the respective growth media, collected by centrifugation (5000 rpm, 5 min), washed twice with physiological solution, counted by plating method, and inoculated in tea. Regarding the native consortium, 40 mL of the artisanal kombucha was centrifuged and the obtained pellet was washed twice with physiological solution, counted, and inoculated in tea. For all trials, the initial microbial concentration was about 10^5^ CFU/mL, determined by plate count after inoculum. The fermentation trials were carried out in triplicate in 200 mL volume and incubated at 28 °C for 14 days.

The different fermentation strategies were labeled with the following codes: KCC (native microbial consortium); TEA (non-inoculated sugared tea used as control for the chemical analysis); Nh (monoculture of *N. hansenii* T7SS-4G1); Zp (monoculture of *Z. parabailii* T7SS-4W1); Bb (monoculture of *B. bruxellensis* T7SB-5W6); NhZp (co-culture of *N. hansenii* T7SS-4G1 + *Z. parabailii* T7SS-4W1), NhBb (co-culture of *N. hansenii* T7SS-4G1 + *B. bruxellensis* T7SB-5W6); NhZpBb (reconstructed microbial consortium with *N. hansenii* T7SS-4G1 + *Z. parabailii* T7SS-4W1 + *B. bruxellensis* T7SB-5W6).

The pH was measured throughout the fermentation process with the pH-meter Crison Basic 20 (Hach-Lange, Barcelona, Spain). In samples inoculated with *N. hansenii* T7SS-4G1, cellulosic matrix production was quantified at the end of fermentation by weighting the samples after water removal at 60 °C for 3 days [29].

### 2.4. High-Performance Liquid Chromatography (HPLC)

To quantify sugars (glucose, fructose), organic acids (acetic, glucuronic, and gluconic acids), and alcohols (glycerol, ethanol) during kombucha tea trials, HPLC analysis was performed at 0, 7, and 14 days of fermentation using the Extrema LC-4000 system (Jasco, Cremella, Italy) coupled with a refractive index detector RI-4030 (Jasco) set to 35 °C. Analytes were separated using a Rezex^TM^ ROA-Organic Acid H + (8%) column (300 × 7.8 mm; Phenomenex, Castel Maggiore, Italy) equipped with a Carbo-H (4 × 3.0 mm) guard column (Phenomenex). The column was maintained at a constant temperature of 80 °C and under an isocratic mobile phase consisting of 5 mM H_2_SO_4_ (Honeywell, Rodano, Italy) at a flow rate of 0.8 mL/min. Before analysis, samples were centrifugated at 6000× *g* for 5 min, filtered with 0.22 µm syringe filters *SPHEROS* (LLG Labware, Meckenheim, Germany), and appropriately diluted with 5 mM H_2_SO_4_. Calibration curves were prepared in a range from 0.05 to 6 g/L.

### 2.5. Analysis of Volatile Compounds

For quantification of alcohols, esters, fatty acids, and benzenoids (except methyl salicylate), the procedure described by [30] was followed, performing a Solid Phase Extraction (SPE) through a BOND ELUT-ENV cartridge (Agilent Technologies. Santa Clara, CA, USA); 50-mL samples of kombucha tea fermentation trials with addition of 100 µL internal standard (2-octanol, 4.2 mg/L in ethanol) were diluted with 50 mL of deionized water. Samples were loaded on the SPE cartridge, previously activated with 20 mL of dichloromethane, 20 mL of methanol, and equilibrated with 20 mL of water. After sample loading, the cartridges were washed with 15 mL of water. Volatile compounds were eluted with 10 mL of dichloromethane and then concentrated under gentle nitrogen stream to 200 μL prior to GC injection.

Free terpenes, norisoprenoids, and methyl salicylate were quantified using Solid Phase Micro Extraction (SPME), following the procedure described by [31]; 5 mL of samples with addition of 5 µL internal standard (2-octanol, 4.2 mg/L in ethanol) were diluted with 5 mL of deionized water and placed into a 20-mL glass vial together with 3 g of NaCl. SPME extraction was performed using a 50/30 μm divinylbenzene–carboxen–polydimethylsiloxane (DVB/CAR/PDMS) fiber (Supelco, Bellafonte, PA, USA) exposed to sample headspace for 60 min at 40 °C.

Volatile sulfur-containing compounds were quantified according to [32] by placing 10 mL of sample with 100 µL of internal standard (DMS-d6, 2 mg/L in ethanol) and 3 g of NaCl into a 20-mL glass vial. SPME extraction was performed using a polydimethylsiloxane-divinylbenzene fibre (PDMS/DVB) (Supelco, Bellafonte, PA, USA) exposed to sample headspace for 30 min.

Gas Chromatography-Mass Spectrometry (GC-MS) analysis was carried out on an HP 7890A (Agilent Technologies, Cernusco sul Naviglio, Italy) gas chromatograph coupled to a 5977B quadrupole mass spectrometer, equipped with a Gerstel MPS3 auto sampler (Müllheim an der Ruhr, Germany). Separation was performed using a DB-WAX UI capillary column (30 m × 0.25 mm, 0.25 μm film thickness, Agilent Technologies) and helium (6.0 grade) as carrier gas at 1.2 mL/min of constant flow rate. The GC oven was programmed as follows: started at 40 °C for 3 min, raised to 230 °C at 4 °C/min and maintained for 20 min. A different program of the GC oven was applied to the vials for quantification of volatile sulfur-containing compounds: started at 35 °C for 5 min, increased to 90 °C at 5 °C/min and then to 260 °C at 15 °C/min, maintained for 2 min. Mass spectrometer was operated in electron ionization (EI) at 70 eV with ion source temperature at 250 °C and quadrupole temperature at 150 °C. Mass spectra were acquired in Selected Ion Monitoring (SIM) mode. Samples were analyzed in random order.

Calibration curves were prepared for all quantification methods. For SPE-GC-MS method, a calibration curve was prepared for each analyte using seven concentration points and three replicates per point in model solution (2% *v*/*v* ethanol, 3.5 g/L tartaric acid, pH 3.0), 100 µL of internal standard 2-octanol (4.2 mg/L in ethanol) was added to each calibration solution, which was then submitted to SPE extraction and GC-MS analysis as described for the samples. For SPME-GC-MS method, a calibration curve was prepared for each analyte using seven concentration points and three replicate solutions per point in model solution. Five µL of internal standard 2-octanol (4.2 mg/L in ethanol) and 100 µL of DMS-d6 (2 mg/L in ethanol) were added to each calibration solution, which was then submitted to SPME extraction and GC-MS analysis as described for the samples. Calibration curves were obtained using Chemstation software (Agilent Technologies) by linear regression, plotting the response ratio (analyte peak area divided by internal standard peak area) against concentration ratio (added analyte concentration divided by internal standard).

### 2.6. Sensory Evaluation

Sensory evaluation of kombucha tea was carried out by means of the sorting task methodology, as described by [33], with slight modifications. Sixteen panelists (eleven males and five females) participated in the sessions. One hour before the test, samples were removed from the 16 °C cold room and 20 mL of each fermentation trial were poured in ISO glasses (https://www.iso.org/standard/9002.html, accessed on 23 August 2022), labeled with 3-digit random codes, and covered by plastic Petri dishes; all samples were served at 22 ± 1 °C, and glasses were presented in random order for each panelist. To assess the reproducibility of the panel, a replicate of one sample was added to the sorting task. Panelists were asked to sort the kombucha samples into groups based on odor similarities exclusively by orthonasal evaluation, with no request to indicate specific odor descriptors, and without limitations on group number.

### 2.7. Statistical Analysis

Data of analytical determinations on GC-MS were compared by three-way analysis of variance (ANOVA), followed by the post-hoc Tukey’s HSD (Honestly Significant Difference) test, with statistical significance threshold set at 95% (*p*-value < 0.05), and Principal Component Analysis (PCA). GC-MS data were averaged, centered, and scaled by compound and hierarchically clustered by Ward’s minimum variance method and Euclidean distance metric with the hclust R [34] function before being plotted by the heatmap.2 function. For the sorting task, data was organized into individual similarity binary matrices (8 × 8 samples; 0 = different and 1 = similar) for each panelist. The dendrogram of hierarchical cluster analysis with the Ward criterion was obtained from a co-occurrence matrix, calculated by the sum of all panelists. Statistical analysis was performed using the package XL-STAT (Addinsoft SARL, Paris, France).

## 3. Results

### 3.1. Kombucha Microbial Population and Collection Set Up

**Figure 1:**
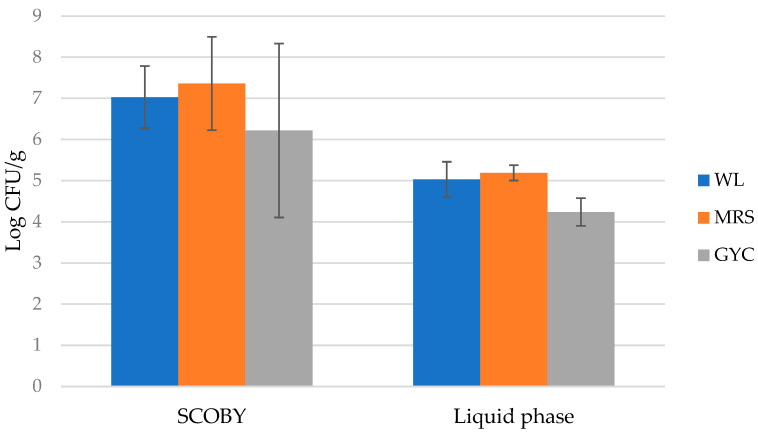
Yeasts and bacterial average microbial load, determined in production tanks of artisanal kombucha tea on different growth media: Wallerstein Lab (WL) agar, MRS and GYC. Bars represent standard deviation.

In the production tanks (T2, T3, and T4), SCOBY samples had higher microbial counts of both yeasts and bacteria than liquid samples, with an average of 6.93 ± 0.50 log CFU/g compared to 4.82 ± 0.51 log CFU/g (Figure 1).

For the same tanks, counts of AAB on GYC medium showed the greatest difference between liquid phase and SCOBY samples (4.24 and 6.22 log CFU/g, respectively), compared to other growth media. From the samples of SCOBY and liquid phase, a total of 64 representative colonies were isolated (33 bacteria, grown on MRS (26) and GYC (7), and 31 yeasts, from WL plates). Most of the yeast isolates (57%) derived from liquid samples, while most of the bacterial isolates (almost 70%) were associated to SCOBY.

The genetic characterization through Rep-PCR analysis with primer (GTG)_5_ led to the detection of 26 unique strains: 13 AAB, 12 yeasts and 1 LAB (Supplementary Figure S1). The 26S rRNA gene sequencing allowed the identification of 8 strains as *B. bruxellensis* (21 isolates), 3 strains as *B. anomalus* (9 isolates), and one strain as *Z. parabailii* (one isolate). As for bacteria, one strain, representative of 11 isolates, was identified as *L. nagelii* through the analysis of 16S rRNA gene sequence, while the sequencing of the gene *dnaK* (coding for the heat shock protein HSP70) led to the identification of 22 AAB isolates belonging to *Komagataeibacter rhaeticus* (3 isolates, 3 strains), *Acetobacter senegalensis* (13 isolates, 5 strains), *N. hansenii* (4 isolates, 3 strains), *Gluconacetobacter entanii* (1 isolate, 1 strain), and *Acetobacter papayae* (1 isolate, 1 strain) (Supplementary Table S1). As shown in Table 1, *B. bruxellensis* and *A. senegalensis* were the most persistent species, recurrent in each analyzed tank, with the highest inter-species diversity (8 and 5 strains, respectively). On the other hand, *Z. parabailii*, *A. papayae*, and *G. entanii* were detected in just one sample, with only one isolate per species.

### 3.2. Tea Fermentation Trials

**Figure 2:**
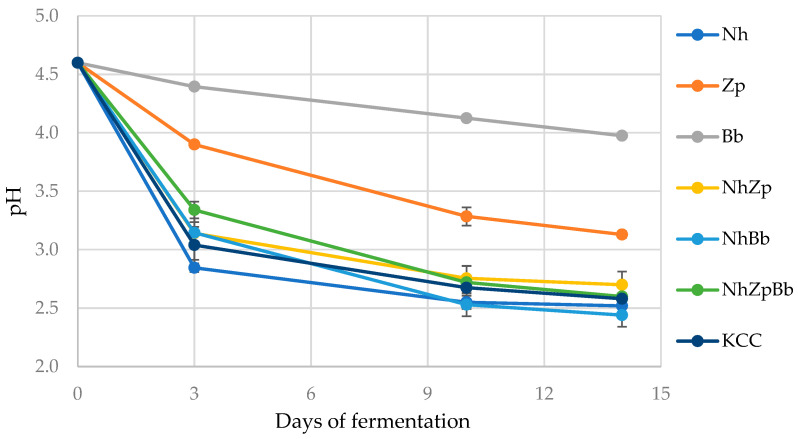
Evolution of pH during kombucha tea fermentation trials. Nh, Zp, Bb, NhZp, NhBb, and NhZpBb: mono- and co-cultures of the strains *N. hansenii* T7SS-4G1 (Nh), *Z. parabailii* T7SS-4W1 (Zp), and *B. bruxellensis* T7SB-5W6 (Bb); KCC: inoculated with the native microbial consortium. Bars represent standard deviation.

Fermentation trials were carried out to investigate the role of selected microorganisms and their interactions in defining the chemical and sensory profile of the studied artisanal kombucha tea. Within the collection, three strains were selected to develop an ad hoc consortium: *N. hansenii* T7SS-4G1 was chosen due to its recognized remarkable capacity to produce extracellular cellulose [5]; *B. bruxellensis* T7SB-5W6 was included for its prevalence in all analyzed samples, along with *Z. parabailii* T7SS-4W1. This yeast strain, even if less represented, was included in the trials for its potential to positively modulate the organoleptic profile of kombucha [25]. The pH was monitored during fermentations to follow the process. Tea was first acidified to pH 4.6 before fermentation, and all mono- and binary co-cultures and microbial consortia caused a further decrease. Two different behaviors were observed (Figure 2). The monocultures Bb and Zp caused a smaller pH change in the first three days, which decreased afterward (3.98 and 3.13 after 14 days, respectively). On the other hand, all the trials with *N. hansenii* (Nh, NhBb, NhZp, NhZpBb) and the native microbial consortium (KCC) substantially lowered the pH in the first days, which was stabilized towards the end, reaching the lowest final values (2.44–2.70).

**Figure 3:**
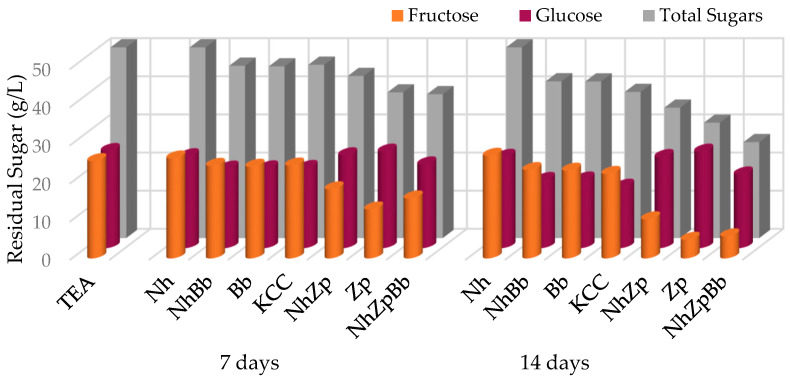
Sugar consumption during kombucha tea fermentation trials. TEA: non-inoculated sugared tea; Nh, Zp, Bb, NhZp, NhBb, and NhZpBb: mono- and co-cultures of the strains *N. hansenii* T7SS-4G1 (Nh), *Z. parabailii* T7SS-4W1 (Zp), and *B. bruxellensis* T7SB-5W6 (Bb); KCC: inoculated with the native microbial consortium.

Sugar concentration measured at the beginning, middle, and end of fermentations showed marked differences among the strain combinations, and three general trends were observed (Figure 3). The monoculture of *N. hansenii* consumed a very low amount of sugar (0.68 ± 0.089 g/L); *B. bruxellensis* had a clear preference for glucose, while *Z. parabailii* was fructophilic and depleted more sugar (9.98 ± 0.21 and 20.79 ± 0.0018 g/L, respectively). In the co-cultures with *Z. parabailii*, a higher sugar consumption (with preference for fructose) was still detected, whereas in the co-culture NhBb and KCC, glucose was mostly consumed, and the total sugar consumption was lower. Overall, the highest sugar consumption was achieved by co-culturing the three selected strains in the reconstructed consortium, depleting more than 75% of fructose and around 25% of glucose.

Regarding the cellulosic matrix production, the highest yield was reached by the Nh monoculture (0.95 ± 0.19 g of cellulose/g of sugars depleted), followed by NhBb (0.042 ± 0.00086 g of cellulose/g of sugars depleted) and NhZp (0.032 ± 0.0050 g of cellulose/g of sugars depleted). As expected, the fermentations with the yeast monocultures did not show any production of cellulosic matrix, while the native microbial consortium and the reconstructed one produced the lowest amounts of cellulosic matrix (Table 2).

As it concerns the production of organic acids, gluconic and glucuronic acids were not detected in all analyzed samples. Meanwhile, the acetic acid content (Table 2) reached the highest score after 14 days of fermentation for the co-culture NhZpBb, which produced 8.37 ± 0.50 g/L. NhZp, NhBb, and KCC showed intermediate values, while the yeast monocultures had the lowest acetic acid production. The monoculture of *N. hansenii* did not produce quantifiable levels.

Glycerol was detected only when yeasts were present, reaching the highest levels in the three fermentations with *Z. parabailii.* When grown in monoculture, *Z. parabailii* produced almost nine times the amount of glycerol compared to *B. bruxellensis* (0.83 and 0.10 g/L, respectively). The same trend was observed for ethanol, which was produced twice as much in Zp compared to Bb monoculture (9.78 and 4.14 g/L, respectively). The co-culture of AAB strain with the yeasts reduced the final level of ethanol produced (1.47 g/L in NhZp and 1.09 g/L in NhBb), concomitant with the increase of acetic acid (2.02 g/L in NhZp and 1.84 g/L in NhBb) (Table 2).

### 3.3. Volatile Organic Compounds

The injection of the pretreated samples of fermented and control sugared tea in the GC-MS allowed for the detection of 35 VOCs above the limit of quantification (Table S2, Supplementary Material). They were divided into six chemical families: alcohols (3), fatty acids (4), esters (7), benzenoids (4), terpenes and norisoprenoids (15), and sulfur-containing compounds (2). The sum of concentrations for each family showed a higher production of alcohols and sulfur-containing compounds in Zp, while Bb increased the levels of benzenoids. The reconstructed consortium with the two yeasts and *N. hansenii*, NhZpBb, notably boosted the accumulation of fatty acids, esters, terpenes, and norisoprenoids, while the native microbial consortium (KCC) reached the highest concentration of fatty acids. The co-cultures NhZp and NhBb generally showed intermediate values, whereas Nh single culture caused nearly the least amount of increase of all molecules, compared with the initial concentrations in the non-fermented sugared tea. Within these VOCs, 25 were produced in significantly different concentrations between fermentation strategies.

**Figure 4:**
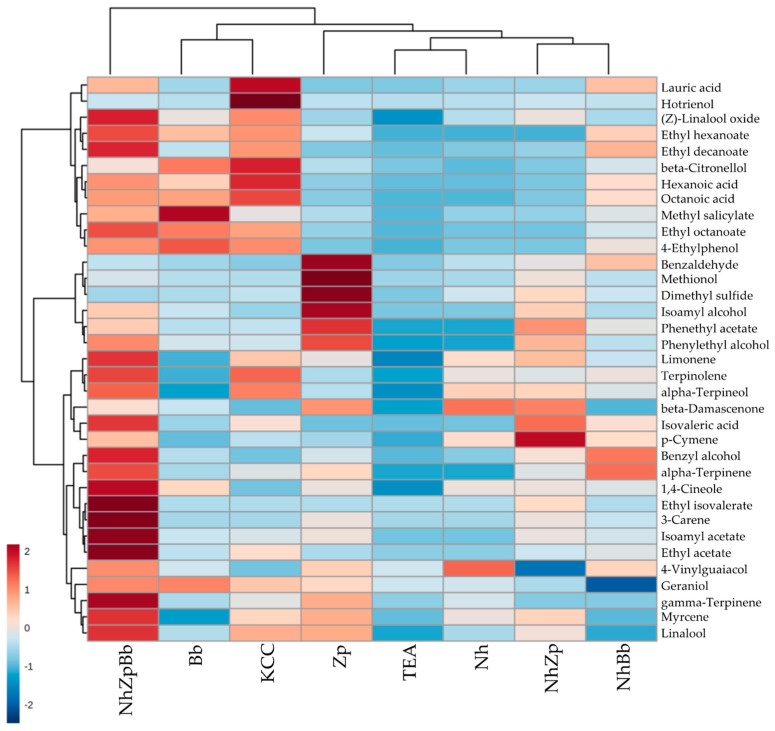
Hierarchical cluster analysis of the relative abundances of VOCs at the end of kombucha tea fermentation trials. TEA: non-inoculated sugared tea; Nh, Zp, Bb, NhZp, NhBb, and NhZpBb: mono- and co-cultures of the strains *N. hansenii* T7SS-4G1 (Nh), *Z. parabailii* T7SS-4W1 (Zp), and *B. bruxellensis* T7SB-5W6 (Bb); KCC: inoculated with the native microbial consortium.

The heat-plot in Figure 4 shows the relative abundances of the single molecules, where the fermentations were clustered according to their similarity on the VOCs profile. NhZpBb caused the general increase of most molecules, especially esters, while the other fermentations stood out for a few key compounds. The native microbial consortium (KCC) produced high concentrations of the medium chain fatty acids, such as caproic (hexanoic), caprylic (octanoic), and lauric (dodecanoic) acids, and of the monoterpenes α-terpineol, β-citronellol, and terpinolene. As for the yeasts’ metabolism, *Z. parabailii* strongly accentuated the levels of isoamyl alcohol, phenylethyl alcohol, phenethyl acetate, benzaldehyde, dimethyl sulfide, and methionol, while *B. bruxellensis* was a high producer of 4-ethylphenol and methyl salicylate. Regarding *N. hansenii*, the only two molecules that reached the highest concentration in the Nh fermentation were 4-vinylguaiacol and β-damascenone—although in both cases the differences were not statistically significant.

**Figure 5:**
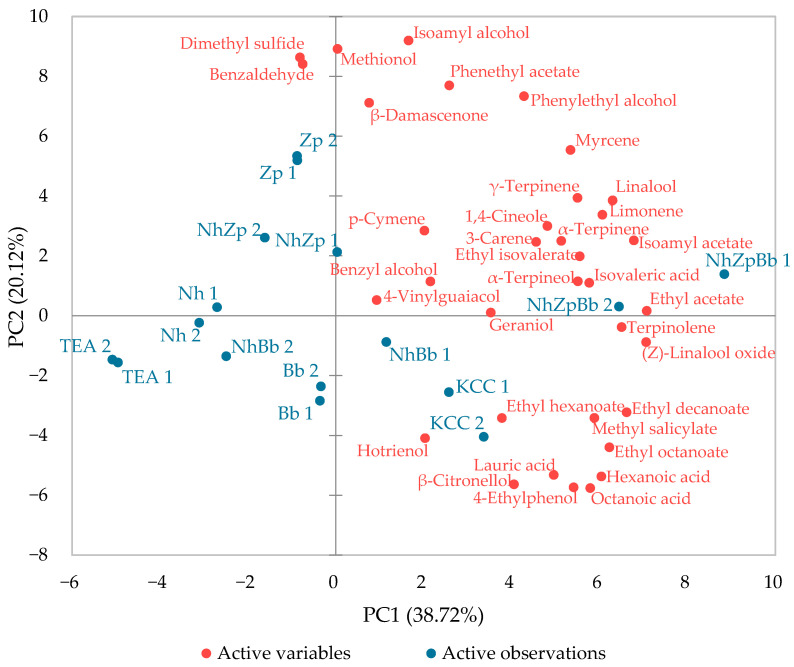
Bi-plot of the Principal Component Analysis with volatile compounds produced during the kombucha tea fermentation trials. The replicates are indicated by the numbers 1 or 2 following the code of each fermentation strategy. TEA: non-fermented sugared tea; Nh, Zp, Bb, NhZp, NhBb, and NhZpBb: mono- and co-cultures of the strains *N. hansenii* T7SS-4G1 (Nh), *Z. parabailii* T7SS-4W1 (Zp), and *B. bruxellensis* T7SB-5W6 (Bb); KCC: inoculated with the native microbial consortium.

All 35 aromatic compounds were subjected to a principal component analysis using the results of the replicates (Figure 5). The first and second components represented in the graph accounted for 58.85% of the total variation (PC1 = 38.72% and PC2 = 20.12%). A good reproducibility was observed, as the replicates were positioned close to each other, except for NhBb1 and NhBb2, which belonged to different quadrants. The main differences highlighted in the heat-plot were also clearly acknowledged in the PCA, in which the non-fermented sugared tea (TEA) fell on the lower left quadrant and NhZpBb, the fermentation with higher overall concentrations, fell on the upper right. The other fermentations, which increased some target molecules, were placed in between. Interestingly, NhZp was closer to Zp, and NhBb closer to Bb. Moreover, the two yeast monocultures, Zp and Bb, denoted their different metabolisms in the production of VOCs.

### 3.4. Sensory Analysis

**Figure 6:**
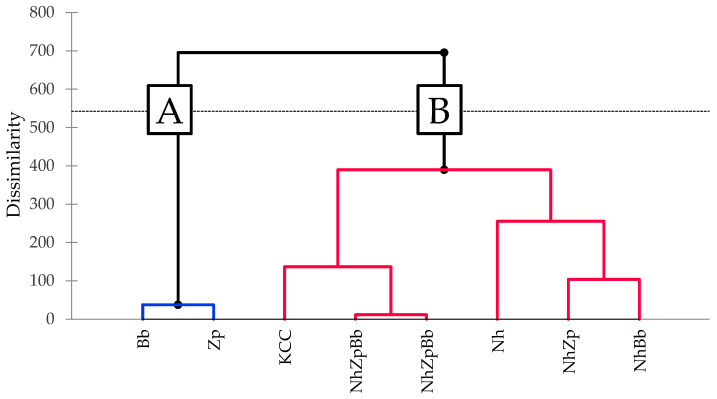
Agglomerative hierarchical clustering of the sorting task scores, at the end of kombucha tea fermentation trials. Nh, Zp, Bb, NhZp, NhBb, and NhZpBb: mono- and co-cultures of the strains *N. hansenii* T7SS-4G1 (Nh), *Z. parabailii* T7SS-4W1 (Zp), and *B. bruxellensis* T7SB-5W6 (Bb); KCC: inoculated with the native microbial consortium. The dashed line indicates the significance threshold.

Sorting task methodology led to formation of two distinctive clusters, A and B (Figure 6). In the first one, *B. bruxellensis* and *Z. parabailii* were grouped based on similarity. Monoculture of *N. hansenii* T7SS-4G1, all co-cultures, and microbial consortia significantly differed from the two yeasts, grouping in cluster B. Panel reproducibility was confirmed by the clustering of NhZpBb, which was analyzed in duplicate, as these two were the most closely related samples. Furthermore, the reconstructed kombucha consortium (NhZpBb) was the most similar to the KCC sample, which represented the kombucha tea fermented with the native microbial consortium.

## 4. Discussion

Kombucha is a fermented beverage resulting from the activity of a complex microbial consortium, in which the contributions of each component have only recently been investigated [10,20,35,36]. In the present study, a microbiota deconstruction/reconstruction approach, combined with analysis of biochemical parameters and fermentation metabolites with organoleptic impact, was applied to guide strain selection and standardize kombucha quality.

Initially, the ecological study carried out on the native microbial consortium of the artisanal kombucha tea allowed us to depict its structure, set up a strain collection, and select distinctive strains to develop an ad hoc consortium. Microbiological analysis highlighted the presence of a large number and diversity of microorganisms, confirming previous results [17], with higher counts of both yeasts and bacteria in SCOBY samples, accounting its ability to protect and trap microorganisms [33]. These data corresponded to those found by [7], who reported, for cellulosic matrix, a minimal count of 7 Log CFU/g, and a lower value for liquid samples (4.4 Log CFU/g) [37].

The molecular analysis of yeast isolates led to the identification of three species: *B. bruxellensis*, *B. anomalus*, and *Z. parabailii*. *Brettanomyces* has been described as the most dominant yeast genus in several black and green tea fermentations [3,7,8]; it is well adapted to harsh environmental conditions, such as low pH and high ethanol concentrations. Generally, *B. anomalus* is prevalent in several kombucha teas [3,7], but in the present study, *B. bruxellensis* was found more abundantly. This aspect could have been related to the intraspecific variability of *B. bruxellensis* in the acetic acid metabolism, which can enable specific strains to outcompete other microbes [38]. This characteristic could be further investigated for the strain *B. bruxellensis* T7SB-5W6, recovered in most of the analyzed samples.

*Zygosaccharomyces* has been frequently detected by culture-dependent and metagenomic analysis among the dominant genera in kombucha [39,40]. However, the species *Z. parabailii*, isolated from the sample T2Sb, has been rarely found in this beverage as it was recovered only in one sample out of 16 samples of different brands and countries [13]. As such, its contribution to kombucha characteristics has not yet been totally unraveled. Interestingly, in one study *Z. parabailii* was found to be co-dominant with *B. bruxellensis* in kombucha obtained with black, green, and rooibos tea [17].

As for AAB, the genera *Komagataeibacter, Novacetimonas,* and *Acetobacter* have been commonly found in kombucha, also by culture-independent methods, revealing that these AAB are dominant and well-adapted to this matrix [3,12,39]. In particular, *N. hansenii* can represent around 20% of AAB in both black and green kombucha teas [7], and is a species of industrial interest known for its application in bacterial cellulose production.

*Liquorilactobacillus nagelii* was the only LAB species with a unique strain detected in this study: a Gram-positive, rod-shaped facultative anaerobe that grows well in an atmosphere enriched with CO_2_ [41]. *L. nagelii* has been previously identified in kombucha samples [7,10], however, the strain *L. nagelii* TLV-4R7 isolated in the present study was not included in the fermentation trials for its very slow growth rate in a preliminary evaluation.

The development of a collection of strains isolated from kombucha and their features was the starting point to design lab-scale tea fermentation trials using different combinations of the strains *N. hansenii* T7SS-4G1, *Z. parabailii* T7SS-4W1, and *B. bruxellensis* T7SB-5W6. All microbial combinations caused a pH decrease, as previously observed in other kombucha fermentation studies [12,42], mainly due to glucose and fructose metabolism during microbial fermentation and related production of organic acids, especially acetic acid. This was dependent on the microbial population, substrate composition, and fermentation time [36]. The rapid pH decrease in the first days was mainly associated with bacterial metabolism [10], as seen in this study and confirmed by the slower decrease observed in yeast monocultures. Additionally, in accordance with the present investigation, previous reports showed a slower pH decrease toward the end of fermentation, with a stabilization around the 10th day thanks to a buffering effect in the fermented medium [10,43,44].

Regarding the different starters inoculated, the Bb monoculture maintained the pH around 4.0 after 10 days of fermentation, which was comparable to the value of 4.17 obtained by [45] after 12 days, even if the starting pH was very different (4.6 against 6.9, respectively). Nh fermentation pH after 10 days was 2.55 and remained stable until the 14th day, which was concordant with the pH value of 2.6 after 7 days found by [46]. Those authors, who used a *N. hansenii* strain isolated from kombucha, stated that a lower pH would inhibit microbial growth and bacterial cellulose production [46]. The consortia KCC and NhZpBb showed a similar pH after 14 days of fermentation (2.58 and 2.60, respectively). These results agreed with [22], who found a pH of 2.4 at the end of fermentation using a reconstructed consortium with *Acetobacter pasteurianus*, *K. xylinus*, and *Z. bailii*. On the contrary, the minimal consortium with *Acetobacter indonesiensis*, *Hanseniaspora valbyensis*, and *B. bruxellensis* proposed by [20] reached a pH value of 4.17 at the end of fermentation, although starting from 6.9.

Sugar depletion was peculiar for each monoculture, reflecting species-specific attitudes. *Z. parabailii* consumed exclusively fructose. This could be attributed to its fructophilic behavior, which was linked to the presence of high-capacity and low-affinity fructose transporters [25]. *B. bruxellensis* also consumed a small amount of fructose, as most *Brettanomyces* strains have the capability to metabolize a wide range of monosaccharides, disaccharides, trisaccharides, and dextrins [38]. In [12], a monoculture of *B. bruxellensis* consumed around 10 g/L of total sugar after 14 days of fermentation, the same value found in the present Bb fermentation, while our strain of *Z. parabailii* consumed two times that amount.

Notable differences in sugar depletion were observed between KCC and NhZpBb, possibly indicating that *Z. parabailii* played a major role in sugar consumption when part of the reconstructed consortium, while its activity was not prevalent in the native microbial consortium. Contrarily, [20] did not find noteworthy differences on the consumption of sugars when testing different strains in mono- and co-cultures to evaluate a proposed minimal consortium.

Chemical parameters, as cellulose, acetic acid, glycerol, and ethanol, were quantified during the fermentation trials. KCC showed significant lower production of those metabolites than NhZpBb, which could be correlated with the lower sugar consumption. In binary combinations, such as NhZp and NhBb, it became clear how yeast-bacterium interactions substantially influenced the chemical composition and were dependent of yeast species due to differential growth rates and metabolic pathways. Different microbial populations and interactions among them might produce kombucha with divergent fermentation kinetics and chemical outcomes, despite encountering the same conditions [2].

The highest amount of cellulose was produced by Nh monoculture, possibly because there was no competition with other microorganisms, and glucose could be exploited to produce cellulosic matrix. As a matter of fact, the more complex consortia KCC and NhZpBb had the lowest yield of cellulose. Only the yeast monocultures could not produce any cellulose at all. No data are currently available for cellulose dry weight production, but [47] reported, for cellulose wet weight, a yield of 3.25 g/g of L-sucrose, using a commercial kombucha as inoculum. In the study of [12], a consistent cellulosic biofilm was only formed with a native kombucha consortium; in the co-cultures of selected strains, the biofilm was not present. Using two different yeast species, *Zygosaccharomyces bisporus* and *B. bruxellensis*, in pairwise combinations with *K. rhaeticus*, the production of biofilm was remarkably higher with *B. bruxellensis* than with *Z. bisporus* [23], thus opposite of this study where more cellulose was recovered from NhZp than NhBb.

The authors of [7] reported acetic acid concentrations between of 7.65–9.18 g/L for green and black tea kombucha, while [10] and [20], investigating reconstructed consortia, found lower levels of 2.41 g/L and 1.05 g/L, respectively. In the present study, values ranged from 0.32 to 8.37 g/L, except for Nh. The lowest end, as expected, was represented by the yeast monocultures, due to the absence of bacterial conversion of ethanol to acetic acid, while the highest production was obtained with the reconstructed consortium NhZpBb. *Novacetimonas* spp. dominate vinegar fermentation, where they are most used, thanks to the resistance to ethanol and acetic acid [48]. Some AAB can use glucose to produce acetic acid, and this compound could be further oxidized via acetyl-CoA synthesis and the TCA cycle [12,22]. Hence, the absence of acetic acid in Nh could be explained by a lack of production or a later consumption of this compound due to oxidation reactions.

Ethanol and glycerol production differed significantly among fermentations, generally reaching higher levels in the presence of Zp, behavior that could be related to the fructophilic character of this species [42,43]. The monoculture Zp produced more ethanol than Bb, and the co-culture NhZp produced more than NhBb. In both co-cultures, a decrease of ethanol was observed as compared with the monocultures, associated with the production of acetic acid from ethanol promoted by Nh [8]. A divergent result was found by [23], who reported a strikingly higher ethanol production by *B. bruxellensis* than *Z. bisporus*.

In NhZpBb, more acetic acid and ethanol were produced than NhZp and NhBb. Possibly, the combined activity of the two yeasts generated more ethanol, thus more substrate was available for the bacterial metabolism to produce acetic acid, also as a response to the stress caused by other strains. Furthermore, acetic acid could stimulate the yeasts to produce more ethanol [12,22], resulting in both metabolites increasing. In previous studies with reconstructed kombucha consortia, other authors found lower ethanol levels than the present investigation, e.g., 0.237 g/L [10] and 0.3 g/L [22].

The ethanol conversion promoted by bacteria in the symbiotic cultures helps to decrease alcohol volume below the legal limits for the classification of non-alcoholic beverages, which is 0.5% *v*/*v* in the USA, Brazil, Australia, and New Zealand, and 1.2% *v*/*v* in the European Union [10,16]. Indeed, in the present investigation, both yeast monocultures exceeded limits, with 0.53% *v*/*v* in Bb and 1.24% *v*/*v* in Zp. Regarding co-cultures, these values dropped, and only the kombucha produced with the reconstructed consortium would have needed to be labeled as alcoholic in the first countries, reaching 0.89% *v*/*v*. Nevertheless, as reported by [16], several commercial kombucha teas presented more than 0.5% *v*/*v* ethanol in the analytical determinations, although these were not correctly labeled.

In the case of glycerol, the dependence on the metabolism of *Z. parabailii* for the generation of this compound was even clearer, as Zp, NhZp, and NhZpBb reached around 0.83 g/L after 14 days of fermentation, while the fermentations without *Z. parabailii* were all below 0.10 g/L. Final concentration of glycerol was reported to be around 0.1–0.5 g/L [9] and 2 g/L [3] in fermented tea, comparable to the present study. Interestingly, *B. bruxellensis* produced less than 1:10 in respect to *Z. parabailii*, and the presence of *N. hansenii* did not influence glycerol production. Glycerol production is normally associated with osmotolerant yeasts, such as the genera *Candida*, *Pichia*, *Schizosaccharomyces*, *Torulaspora*, and *Zygosaccharomyces* [3]. Some *Novacetimonas* (former *Komagataeibacter)* prefer the oxidation of ethanol, whereas *Gluconobacter* in general favor the oxidation of glycerol, glucose, gluconic acid, and sorbitol over ethanol [48]. Glycerol has been related to a positive influence on body and viscosity in wine and can increase the sweetness and smoothness of the beverage [49].

Volatilome profiles depict each strain contribution to microbial consortia, linking chemical parameters to possible sensory repercussions. Alcohols (e.g., isoamyl alcohol, phenylethyl alcohol), fatty acids (e.g., hexanoic acid, octanoic acid), esters (e.g., ethyl acetate, isoamyl acetate), and terpenes (e.g., linalool, β-citronellol) production were well correlated with yeast activity (*Z. parabailii* and *B. bruxellensis*), as also reported in [20]. Some molecules were also differentially produced between the two yeast species, since they could take different routes for nutrient utilization and production of secondary metabolites, even though central carbon metabolism was mostly conserved [50]. Zp monoculture produced almost six times more isoamyl alcohol compared to Bb and three times more phenylethyl alcohol, confirmed in co-culture, in which the contribution of *Z. parabailii* was crucial. Phenylethyl alcohol was found in greater proportion in kombucha with higher ethanol and glycerol [3], which was also observed in the present study, associated with the presence of *Z. parabailii*.

Fermentations with *B. bruxellensis* produced the highest overall concentration of fatty acids, which could be related to the fact that other kombucha produced in the absence of this species had the lowest level of acids [3]. Interestingly, Nh showed a comparable lauric acid concentration to Bb (105.50 and 130.00 µg/L, respectively) and the production was greatly enhanced when they were co-cultured, reaching 701.55 in NhBb and 742.50 µg/L in NhZpBb, but that was still less than half the amount reached in KCC sample (1472.00 µg/L). Lauric acid has a strong antimicrobial activity, as it is effective against bacteria that are prevalent in overweight people, thus helping to control obesity [4]. In the case of isovaleric acid, the concentrations in all co-cultures were superior to those found in the monocultures, also highlighting the synergistic effects of the microbial interactions. Moreover, this molecule is commonly produced by *B. bruxellensis*; however, it was coupled with unpleasant acidity in kombucha, and described as earthy, medicinal, and sweaty [19,20]. Fatty acids are generally released in wine during autolysis of yeasts and could be involved in the yeasty aroma of kombucha, and alongside higher alcohols, they promote aromas of vinegar, apple juice, and exotic fruits [20].

Almost all esters, terpenes, and norisoprenoids reached their highest concentrations in the kombucha fermented with the consortia KCC and NhZpBb. Those are important aromatic compounds in fermented beverages, usually associated with pleasant floral and fruity aromas [51,52]. Esters enhance tea and white fruit odor perceptions in kombucha [20]. Ethyl acetate production was enhanced when all strains were co-cultured, reaching 51.30 µg/L, while both pairwise yeast-bacteria combinations failed to reach more than 10 µg/L. This effect could be explained by yeast capacity of converting ethanol into ethyl acetate, where yeast synergy is required to obtain a higher amount of this compound [53]. The synergistic effect was observed for the other esters as well, except for ethyl octanoate and phenethyl acetate, which were strongly associated with *B. bruxellensis* and *Z. parabailii*, respectively. The first also reached the highest concentration of the precursor octanoic acid, while the latter produced the most phenylethyl alcohol—as expected, considering the yeast metabolic pathways of fatty acid ethyl esters and acetate esters, respectively [52].

Regarding terpenes, the synergy of multiple species increased their levels, but some individual contributions of the selected strains were clearly acknowledged. *B. bruxellensis* could be associated with β-citronellol and *Z. parabailii* with linalool. NhZpBb co-culture showed the highest linalool increase (11.27 µg/L), where the main yeast responsible was *Z. parabailii* which in monoculture reached 8.17 µg/L, almost doubling the concentration found for *B. bruxellensis* (4.14 µg/L). Linalool content increased in honey byproduct fermentation by a microbial consortium that included *Z. bailii* [54]. Although terpenes have been considered varietal aromas and *de novo* biosynthesis of terpenes by *Saccharomyces* and non-*Saccharomyces* yeasts was normally neglected, some *S. cerevisiae* and *Hanseniaspora uvarum* wild strains produced up to 4 µg/L linalool and 1 µg/L β-citronellol in a chemically defined must-like medium [55].

*B. bruxellensis*, in monoculture, produced the highest concentrations of the benzenoids 4-ethylphenol and methyl salicylate, while single *Z. parabailii* was the biggest producer of the sulfur-containing compounds dimethyl sulfide and methionol, and benzaldehyde. Methyl salicylate (spicy, wintergreen) and benzaldehyde (cherry, almond), as linalool and β-citronellol, are mainly released from bound glycosides in fermented beverages by yeast-derived glycosidase enzymes, and they are all important contributors to the flavor of tea [56,57]. *Brettanomyces* spp. and *Zygosaccharomyces* spp. were considered to have low β-glucosidase activity [19], albeit a β-glucosidase isolated from *B. anomalus* enhanced methyl salicylate and linalool in forest fruit milk [58]. As for 4-ethylphenol, its formation from p-coumaric acid has been exhaustively described in *B. bruxellensis*, but elevated concentrations can give aromas of horse sweat, barnyards, and medicine [59]. Dimethyl sulfide and methionol, with aromas of cabbage, truffle, and boiled potato, are volatile sulfur-containing compounds produced during fermentation by yeast metabolism, highly dependent of species and strain. Concentrations of these two compounds were strikingly higher in Zp, but we did not find any study about volatile sulfur-containing compounds metabolism, either with *Zygosaccharomyces* spp. or *Brettanomyces* spp., despite extensive research with *S. cerevisiae* and other non-conventional yeasts [60,61] To the best of our knowledge, this was the first study ever to consider the presence of terpenes, norisoprenoids, and sulfur-containing compounds in kombucha, even though these molecules and their precursors were already described in tea [56].

The ability of *Z. parabailii* to produce alcohols and sulfur-containing compounds was compressed in the reconstructed consortia, while the production of fatty acids and benzenoids by *B. bruxellensis* was maintained or augmented when co-inoculated with the other strains. Accumulation of esters was led by yeast metabolism and not affected by the yeast-bacteria interaction, but a synergistic effect was seen in the consortium comprising the two yeast strains. For terpenes and norisoprenoids, the concentration in the reconstructed consortium was possibly a contribution of the three strains. These changes, associated with the microbial interactions, could be related with modifications to oxygen access due to pellicle formation, alterations of pH, competition for substrates, and cross-feeding of metabolites [10,45].

Sensory analysis was conducted to evaluate both mono- and co-cultures by sorting task method. The clustering was not identical to the VOCs profile, but some trends were still visible, and certain correlations could be suggested between the chemical analysis and the sensory outcomes. Peculiar VOCs production of Bb and Zp affected panelists’ evaluation, showing a distinctive profile from other samples. The separation of the monocultures from the other fermentations was most likely defined by the much lower concentration of acetic acid. The authors of [36] defined, in kombucha, an odor perception threshold of 0.21 g/L for acetic acid, whereas it became pungent in concentrations above 1.25 g/L. Therefore, acetic acid could be theoretically perceived in all fermentations, but it only became pungent in the co-cultures and microbial consortia.

The two binary co-cultures clustered together in both analyses; however, in sensory analysis, they clustered closer to the bacterial monoculture than to the yeasts. This could suggest that, although yeast metabolism was more influent in the carbon metabolism and production of secondary metabolites, participation of the bacteria was more influential in the sensory response. The reconstructed consortium (NhZpBb) was the most closely related to KCC, possibly because these two samples had the highest concentrations of fatty acids, which can mask the aromatic notes of tea esters and ketones [20]. Nevertheless, the presence of fatty acids precursors could contribute to the biosynthesis of their respective ethyl esters, thus modulating the aromatic equilibrium [62]. Indeed, the higher levels of acetic, hexanoic, and octanoic acids in NhZpBb and KCC were accompanied by increased ethyl acetate, ethyl hexanoate, and ethyl octanoate.

These results showed that the combination of the three selected strains was enough to mimic a more complex kombucha consortium made up of more species, providing a possible way to standardize commercial kombucha production. Satisfactory products fermented by a new tailor-made consortium, with similarities to classical kombucha obtained by inoculation of the established consortium from a previous batch, were also observed in previous sensory analysis [20,36].

## 5. Conclusions

This study explored the features of a microbial consortium used for the artisanal production of kombucha tea, through microbiological, chemical, and sensory analysis, providing a better understanding of the role of distinctive microorganisms. The isolation and taxonomic identification of yeasts and bacteria revealed the ample biological and functional diversity of the analyzed microbial community. It was composed of at least 12 yeast and 14 bacterial strains belonging to nine species. The microbiota deconstruction–reconstruction approach applied here allowed the selection of a restricted number of microorganisms which performed important functions in a wider community, and to disentangle the metabolic activity of each selected strain. The capability of *N. hansenii* T7SS-4G1 to produce acetic acid and cellulosic matrix, essential compounds for the kombucha taste and characteristics, confirmed the role usually attributed to AAB in this beverage. *B. bruxellensis* T7SB-5W6 and *Z. parabailii* T7SS-4W1 showed synergistic activity, boosting the concentration of esters and terpenes, which are responsible for the fruity and floral aromas usually desired in fermented beverages.

In conclusion, this research reconstructed a microbial association capable of producing a fermented beverage with the typical sensory features of the studied kombucha, simplifying the native consortium by using only three strains instead of the 26 isolated from the artisanal scale production. Thus, we demonstrated the possibility of standardizing a fermentation process to obtain a final product with desirable (and more predictable) aromatic and sensory qualities. To confirm these results, a fermentation scaling-up using the reconstructed consortium should be carried out in future research. Moreover, ad hoc consortia could be used to develop both sensory and chemical parameters, focusing on beneficial compounds through the enhancement of the production of key metabolites.

## Figures and Tables

**Table 1:** Distribution of yeasts and bacteria strains/isolates per sample (S: SCOBY and L: liquid phase; a, b: two different samples) in tanks (T1–T5) of artisanal kombucha tea. For each genetic profile, a two-letter distinctive code is reported.

Species	N° of Strains/ N° of Isolates Per Sample (Genetic Profiles Code)										
Strain/Isolates	T1S	T2L	T2Sa	T2Sb	T3L	T3S	T4L	T4S	T5L	T5Sa	T5Sb
*B. bruxellensis*	1/2 (bc)	2/3 (bc, bg)	2/3 (bc, bf)	1/1 (bc)	1/1 (be)	2/2 (bf, bh)	2/2 (bd, bf)	1/2 (ba)	1/1 (bc)	2/2 (bb, bc)	1/2 (bc)
*B. anomalus*		1/1 (aa)	1/2 (aa)		1/1 (aa)		2/2 (ab, ac)	1/1 (aa)	1/1 (ab)		1/1 (ba)
*Z. parabailii*				1/1 (za)							
*K. rhaeticus*			1/1 (kc)							2/2 (ka, kb)	
*L. nagelii*	1/1 (la)		1/2 (la)					1/5 (la)		1/1 (la)	1/2 (la)
*N. hansenii*			1/1 (hb)	1/1 (hc)					1/1 (ha)	1/1 (ha)	
*A. senegalensis*	2/3 (sa, sd)	1/1 (se)	2/2 (sa, sd)			1/2 (sc)		3/3 (sa, sc, sd)		1/1 (sb)	1/1 (sc)
*A. papayae*	1/1 (pa)										
*G. entanii*				1/1 (ga)							

Genetic profiles of strains associated with each species: *B. bruxellensis,* ba, bb, bc, bd, be, bf, bg, bh; *B. anomalus*, aa, ab ac; *K. rhaeticus*, ka, kb, kc; *L. nagelii*, la; *N. hansenii*, ha, hb, hc; *A senegalensis*, sa, sb, sc, sd, se; *A. papayae*, pa; *G. entanii*, ga.

**Table 2:** Concentration (g/L) of glycerol, acetic acid, ethanol, and cellulose dry weight in fermentation trials. Nh, Zp, Bb, NhZp, NhBb, and NhZpBb: mono- and co-cultures of the strains *N. hansenii* T7SS-4G1 (Nh), *Z. parabailii* T7SS-4W1 (Zp), and *B. bruxellensis* T7SB-5W6 (Bb); KCC: inoculated with the native microbial consortium.

Trials	Glycerol		Acetic Acid		Ethanol		Cellulose Dry Weight at 14 Days
	7 Days	14 Days	7 Days	14 Days	7 Days	14 Days	
Nh	-	-	-	-	-	-	0.65 ± 0.21
Zp	0.54 ± 0.01	0.83 ± 0.03	0.31 ± 0.04	0.32 ± 0.01	4.79 ± 1.35	9.78 ± 0.29	-
Bb	0.05 ± 0.00	0.10 ± 0.00	0.37 ± 0.00	0.43 ± 0.02	2.46 ± 0.01	4.14 ± 0.03	-
NhZp	0.50 ± 0.06	0.77 ± 0.18	2.02 ± 0.96	4.62 ± 2.92	1.47 ± 0.66	3.66 ± 0.99	0.54 ± 0.11
NhBb	0.04 ± 0.01	0.10 ± 0.01	1.84 ± 0.53	3.51 ± 0.39	1.09 ± 0.47	1.51 ± 0.19	0.42 ± 0.03
NhZpBb	0.59 ± 0.05	0.87 ± 0.12	3.39 ± 0.66	8.37 ± 0.50	3.33 ± 0.69	7.00 ± 1.10	0.31 ±0.02
KCC	N.D.	0.08 ± 0.35	1.04 ± 0.35	4.27 ± 1.05	1.49 ± 0.43	1.94 ± 0.45	0.20 ± 0.04

Data are represented as mean values ± standard deviation.

## Data Availability

The data presented in this study are available on request from the corresponding author.

## Supplementary Materials

The following supporting information can be downloaded at: https://www.mdpi.com/article/10.3390/foods11193045/s1, Figure S1: Rep-PCR profile clustering of yeasts (A) and bacteria (B) isolated from artisanal kombucha tea. Representative isolates of clusters (curly brackets), identified by *, were chosen for identification. The red bar represents the threshold similarity percentage upon which the clustering was made; Table S1: Identification of isolates based on *dnaK* and 16S rRNA gene sequences for acetic acid bacteria and lactic acid bacteria, respectively, and 26S rRNA gene sequences for yeasts. A two-letter distinctive code is reported for each genetic profile; Table S2: Volatile organic compounds (μg/L) in the fermented and non-fermented tea. TEA: non-inoculated sugared tea; Nh, Zp, Bb, NhZp, NhBb, and NhZpBb: mono- and co-cultures of the strains *N. hansenii* T7SS-4G1 (Nh), *Z. parabailii* T7SS-4W1 (Zp), and *B. bruxellensis* T7SB-5W6 (Bb); KCC: inoculated with the native microbial consortium.
