# Supplementary material for: Reconstruction of Simplified Microbial Consortia to Modulate Sensory Quality of Kombucha Tea
Source: Foods. 2022 Sep 30;11(19):3045. doi: 10.3390/foods11193045 (PMC9563716; doi:10.3390/foods11193045)
Supplement: Supplementary file 1 [file foods-11-03045-s001.zip › foods-1919498-supplementary.pdf]

Reconstruction of simplified microbial consortia to modulate sensory quality of kombucha tea

Nicola Ferremi Leali[†], Renato L. Binati [†], Francesco Martelli, Veronica Gatto, Giovanni Luzzini, Andrea Salini, Davide Slaghenaufi, Salvatore Fusco, Maurizio Ugliano, Sandra Torriani * and Elisa Salvetti

Supplementary Figure S1. Rep-PCR profile clustering of yeasts (A) and bacteria (B) isolated from artisanal kombucha tea. Representative isolates of clusters (curly brackets), identified by *, were chosen for identification. The red bar represents the threshold similarity percentage upon which the clustering was made.

(A)

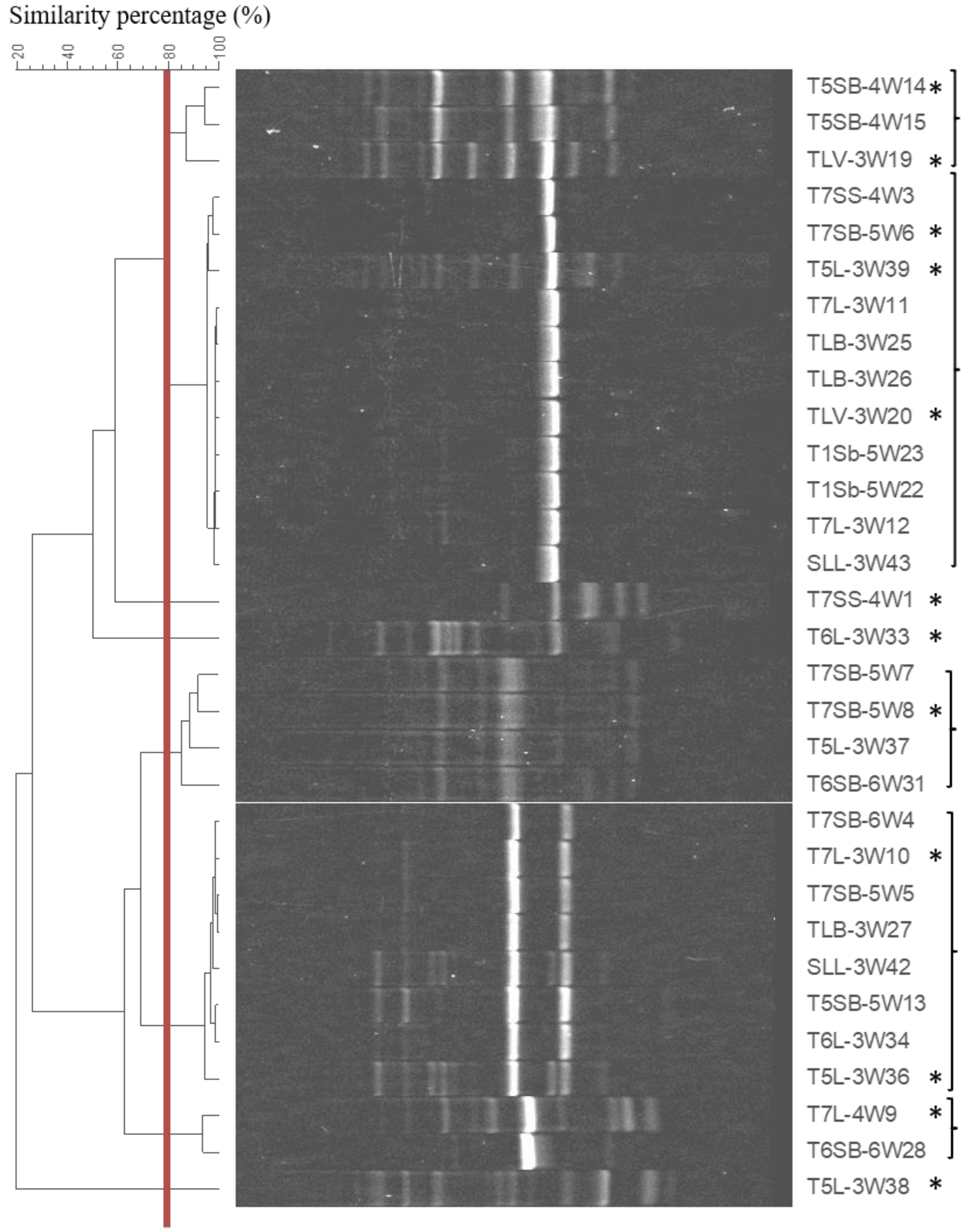

(B)

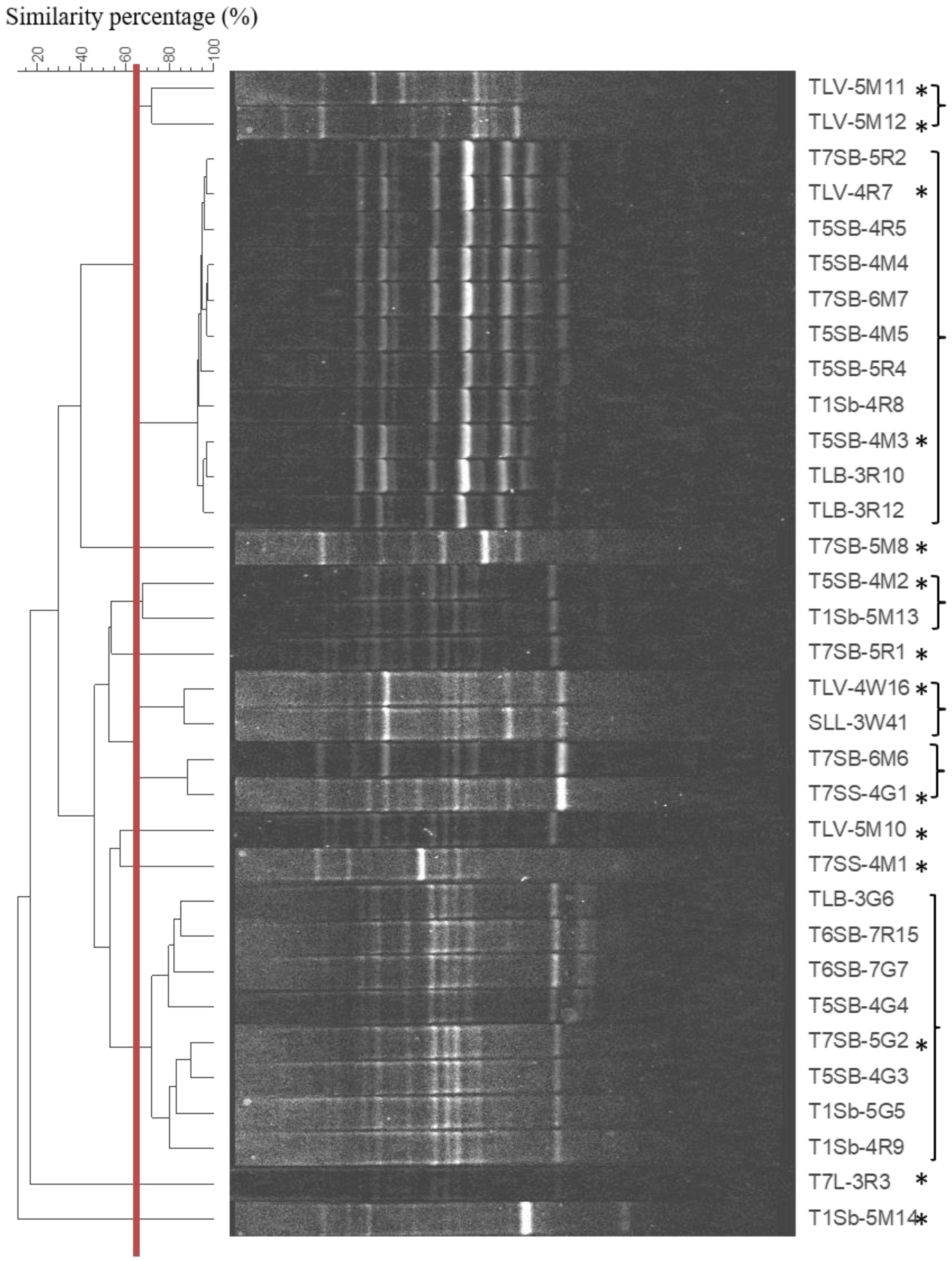
Similarity percentage (%)
20
40
60
80
100
TLV-5M11 *
TLV-5M12 *
T7SB-5R2
TLV-4R7 *
T5SB-4R5
T5SB-4M4
T7SB-6M7
T5SB-4M5
T5SB-5R4
T1Sb-4R8
T5SB-4M3 *
TLB-3R10
TLB-3R12
T7SB-5M8 *
T5SB-4M2 *
T1Sb-5M13
T7SB-5R1 *
TLV-4W16 *
SLL-3W41
T7SB-6M6
T7SS-4G1 *
TLV-5M10 *
T7SS-4M1 *
TLB-3G6
T6SB-7R15
T6SB-7G7
T5SB-4G4
T7SB-5G2 *
T5SB-4G3
T1Sb-5G5
T1Sb-4R9
T7L-3R3 *
T1Sb-5M14 *

Supplementary Table S1. Identification of isolates based on *dnaK* and 16S rRNA gene sequences for acetic acid bacteria and lactic acid bacteria, respectively, and 26S rRNA gene sequences for yeasts. A two-letter distinctive code is reported for each genetic profile.

Isolate	Profile code	Closest relative	Accession N° closest relative	Identity (%)	Alignment*	Accession Number
Acetic acid bacteria						
T1SB-5M14	ap	*Acetobacter papayae* JCM 25143 T	NZ_BAIN00000000.1	99.70	328/329	OP499955
T5SB-4M2	aa	*Acetobacter senegalensis* LMG 23690 T	NZ_LHZU00000000.1	98.48	324/329	OP499956
TLV-5M10	ab	*Acetobacter* senegalensis LMG 23690 T	NZ_LHZU00000000.1	98.18	323/329	OP499960
T7SB-5G2	ad	*Acetobacter senegalensis* LMG 23690 T	NZ_LHZU00000000.1	98.48	324/329	OP499957
		Acetobacter tropicalis LMG 19825 T	NZ_LHZQ00000000.1	96.96	324/329	
T7SB-5R1	aa	*Acetobacter senegalensis* LMG 23690 T	NZ_LHZU00000000.1	98.48	324/329	OP499958
		Acetobacter tropicalis LMG 19825 T	NZ_LHZQ00000000.1	96.96	319/329	
T7L-3R3	ae	*Acetobacter senegalensis* LMG 23690 T	NZ_LHZU00000000.1	98.48	324/329	OP499959
		Acetobacter tropicalis LMG 19825 T	NZ_LHZQ00000000.1	96.96	319/329	
T7SS-4M1	ga	*Gluconoacetobacter entanii* LTH 4560T	NZ_NKUF00000000.1	98.78	325/329	OP499952
TLV-5M12	kb	*Komagataeibacter rhaeticus* LMG 22126 T	NZ_NKTZ00000000.1	98.78	324/328	OP499951
TLV-5M11	ka	*Komagataeibacter rhaeticus* LMG 22126 T	NZ_NKTZ00000000.1	99.39	326/328	OP499950
T7SB-5M8	kc	*Komagataeibacter rhaeticus* LMG 22126 T	NZ_NKTZ00000000.1	99.39	327/329	OP499949
TLV-4W16	ha	*Novacetimonas hansenii* NBRC 14820 T	NZ_BJNN00000000.1	99.09	325/328	OP499954
T7SS-4G1	hc	*Novacetimonas hansenii* NBRC 14820 T	NZ_BJNN00000000.1	99.09	325/328	OP499953
Lactic acid bacteria						
T5SB-4M3	la	*Liquorilactobacillus nagelii* DSM 13675 T	NZ_AZEV00000000.1	99.1	1010/1019	OP302744
TLV-4R7	la	*Liquorilactobacillus nagelii* DSM 13675 T	NZ_AZEV00000000.1	99.9	1019/1020	OP302745
Yeasts						
T7L-3W10	aa	*Brettanomyces anomalus* CBS 77	KY107592	99.4	534/537	OP302791
T5L-3W36	ab	*Brettanomyces anomalus* CBS 77	KY107592	99.6	534/536	OP302792
T5L-3W38	ac	*Brettanomyces anomalus* CBS 77	KY107592	100.0	453/453	OP302793
T7SB-5W6	bc	*Brettanomyces bruxellensis* CBS 74	KY107614	99.8	540/541	OP302794
T7SB-5W8	bf	*Brettanomyces bruxellensis* CBS 74	KY107614	99.8	540/541	OP302795
T7L-4W9	bg	*Brettanomyces bruxellensis* CBS 74	KY107614	99.8	540/542	OP302796
T5SB-4W14	ba	*Brettanomyces bruxellensis* CBS 74	KY107614	99.6	539/541	OP302797
TLV-3W19	bb	*Brettanomyces bruxellensis* CBS 74	KY107614	99.8	540/541	OP302798
TLV-3W20	bc	*Brettanomyces bruxellensis* CBS 74	KY107614	99.8	540/541	OP302799
T6SB-6W28	bh	*Brettanomyces bruxellensis* CBS 74	KY107614	99.8	540/541	OP302800
T6L-3W33	be	*Brettanomyces bruxellensis* CBS 74	KY107614	100.0	458/458	OP302801
T5L-3W39	bd	*Brettanomyces bruxellensis* CBS 74	KY107614	99.8	540/541	OP302802
T7SS-4W1	zp	*Zygosaccharomyces parabailii* ATCC 60483	CP019493	99.1	535/540	OP302803

* Alignment refers the comparison between the query sequence and the reference sequence.

.

Supplementary Table S2. Volatile organic compounds (μg/L) in the fermented and non-fermented tea. TEA: non-inoculated sugared tea; Nh, Zp, Bb, NhZp, NhBb, and NhZpBb: mono- and co-cultures of the strains *N. hansenii* T7SS-4G1 (Nh), *Z. parabailii* T7SS-4W1 (Zp), and *B. bruxellensis* T7SB-5W6 (Bb); KCC: inoculated with the native microbial consortium.

Compound	IUPAC name	OD	Fermentation trials								p-value
			TEA	Nh	Zp	Bb	NhZp	NhBb	NhZpBb	KCC	
Alcohols											
Isoamyl alcohol	3-methylbutan-1-ol	Roasted, spicy, sweet	19.34 ± 2.66c	200.01 ± 165.44c	27780.34 ± 1333.48a	4769.81 ± 421.89bc	11229.98 ± 6532.80b	3108.07 ± 2703.13bc	11664.47 ± 1047.96b	1912.00 ± 381.16bc	<0.0001
Phenylethyl alcohol	2-phenylethanol	Sweet, floral, fresh	10.50 ± 0.53c	37.01 ± 10.22c	1273.04 ± 42.84a	483.89 ± 24.57bc	879.73 ± 407.11ab	410.42 ± 260.57bc	1063.56 ± 15.13ab	474.64 ± 115.99bc	0.001
Benzyl alcohol	phenylmethanol	Floral, rose, phenolic	0.20 ± 0.01a	0.44 ± 0.41a	1.01 ± 0.14a	0.77 ± 0.16a	1.30 ± 1.68a	2.45 ± 1.08a	3.08 ± 4.17a	0.32 ± 0.02a	0.601
∑ *ALCOHOLS*			*30.03*	*237.45*	*29054.39*	*5254.46*	*12111.00*	*3520.93*	*12731.11*	*2386.95*	
Fatty acids											
Isovaleric acid	3-methylbutanoic acid	Sour, sweaty, cheesy	3.66 ± 1.15c	49.08 ± 1.10bc	28.50 ± 1.08bc	272.47 ± 13.82bc	1892.04 ± 864.97ab	917.20 ± 970.45abc	2229.12 ± 268.23a	890.92 ± 128.59abc	0.007
Hexanoic acid	hexanoic acid	Sour, fatty, sweaty	8.22 ± 1.94c	16.15 ± 1.75c	84.34 ± 15.47c	413.37 ± 14.10bc	44.39 ± 0.98c	354.24 ± 293.76bc	601.66 ± 2.16ab	850.00 ± 58.54a	0.000
Octanoic acid	octanoic acid	Fatty, waxy, rancid	133.36 ± 13.54d	154.56 ± 17.37d	600.26 ± 12.98d	2931.71 ± 14.42b	456.63 ± 23.67d	1983.56 ± 611.73c	3022.25 ± 106.26b	3997.11 ± 45.64a	<0.0001
Lauric acid	dodecanoic acid	Fatty, coconut	0.00 ± 0.00c	105.50 ± 0.71c	0.00 ± 0.00c	130.00 ± 12.73c	108.50 ± 13.44c	701.55 ± 45.23b	742.50 ± 20.51b	1472.00 ± 186.68a	<0.0001
∑ *FATTY ACIDS*			*145.23*	*325.29*	*713.10*	*3747.55*	*2501.55*	*3956.54*	*6595.52*	*7210.03*	
Esters											
Ethyl acetate	ethyl acetate	Ethereal, fruity, sweet	0.00 ± 0.00c	0.0 ± 0.03c	3.62 ± 0.36c	5.66 ± 0.31bc	7.51 ± 2.98bc	9.43 ± 1.12bc	51.30 ± 5.34a	15.32 ± 4.33b	<0.0001
Ethyl isovalerate	ethyl 3-methylbutanoate	fruity sweet apple	0.00 ± 0.00b	0.00 ± 0.00b	0.00 ± 0.00b	0.00 ± 0.00b	0.44 ± 0.62b	0.00 ± 0.00b	1.47 ± 0.37a	0.00 ± 0.00b	0.004
Ethyl hexanoate	ethyl hexanoate	Sweet, fruity, pineapple	0.00 ± 0.00b	0.00 ± 0.00b	1.07 ± 0.32ab	2.03 ± 0.02ab	0.00 ± 0.00b	1.82 ± 0.67ab	3.07 ± 0.69a	2.45 ± 1.07a	0.002
Ethyl octanoate	ethyl octanoate	Fruity, winey, waxy	2.12 ± 0.03c	2.39 ± 0.11c	2.86 ± 0.48c	6.71 ± 0.17a	2.41 ± 0.06c	3.78 ± 1.39bc	7.42 ± 0.70a	6.01 ± 0.98ab	0.000
Ethyl decanoate	ethyl decanoate	Sweet, waxy, fruity	0.83 ± 0.13c	1.07 ± 0.06c	1.08 ± 0.17c	1.87 ± 0.01c	1.32 ± 0.05c	3.69 ± 0.11b	5.73 ± 0.49a	4.18 ± 1.05ab	<0.0001
Isoamyl acetate	3-methylbutyl acetate	Sweet, fruity	0.00 ± 0.00c	0.00 ± 0.00c	18.99 ± 0.83b	13.31 ± 0.27bc	18.39 ± 1.05b	14.76 ± 3.59b	62.04 ± 8.75a	15.30 ± 2.86b	<0.0001

Phenethyl acetate	2-phenylethyl acetate	Floral, rose, sweet	0.00 ± 0.00c	0.00 ± 0.00c	41.56 ± 2.87a	13.04 ± 0.04bc	31.84 ± 17.56ab	17.66 ± 1.09abc	24.43 ± 2.09abc	14.17 ± 0.45bc	0.002
∑ *ESTERS*			*2.95*	*3.49*	*69.17*	*42.61*	*61.90*	*51.13*	*155.44*	*57.42*	
Benzenoids											
4-ethylphenol	4-ethylphenol	Phenolic, smoky	0.00 ± 0.00c	3.56 ± 0.04c	3.63 ± 0.03c	30.58 ± 0.06a	3.64 ± 0.06c	13.66 ± 5.65b	24.74 ± 0.38a	25.78 ± 0.43a	<0.0001
4-vinylguaiacol	4-ethenyl-2-methoxyphenol	Spicy, peppery	0.06 ± 0.01a	0.08 ± 0.01a	0.07 ± 0.01a	0.06 ± 0.01a	0.03 ± 0.04a	0.06 ± 0.01a	0.08 ± 0.01a	0.05 ± 0.01a	0.230
Benzaldehyde	benzaldehyde	Almond, fruity	0.77 ± 0.08c	1.23 ± 0.05bc	4.46 ± 0.24a	1.02 ± 0.06bc	1.64 ± 0.22bc	2.37 ± 0.84b	1.29 ± 0.30bc	0.80 ± 0.13c	<0.0001
Methyl salicylate	Methyl 2-hydroxybenzoate	Wintergreen, minty	0.17 ± 0.00c	0.28 ± 0.01bc	0.33 ± 0.06bc	1.11 ± 0.01a	0.28 ± 0.09bc	0.43 ± 0.16bc	0.69 ± 0.25ab	0.45 ± 0.03bc	0.001
∑ *BENZENOIDS*			*1.00*	*1.58*	*4.86*	*32.76*	*1.94*	*16.52*	*26.79*	*27.07*	
Terpenes + Norisoprenoids											
1,4-cineole	1-methyl-4-propan-2-yl-7-oxabicyclo [2.2.1] heptane	Cooling, pine, minty	0.08 ± 0.00a	0.11 ± 0.02a	0.11 ± 0.01a	0.11 ± 0.00a	0.11 ± 0.01a	0.10 ± 0.00a	0.14 ± 0.06a	0.09 ± 0.00a	0.366
3-carene	3,7,7-trimethylbicyclo [4.1.0] hept-3-ene	Citrus, terpenic, herbal	0.00 ± 0.00a	0.00 ± 0.00a	0.01 ± 0.01a	0.00 ± 0.00a	0.01 ± 0.01a	0.003 ± 0.00a	0.03 ± 0.02a	0.00 ± 0.00a	0.167
(Z)-linalool oxide	2-(5-ethenyl-5-methyloxolan-2-yl) propan-2-ol	Floral, herbal	0.60 ± 0.13b	1.34 ± 0.16ab	1.23 ± 0.05ab	1.67 ± 0.39ab	1.68 ± 0.46ab	1.28 ± 0.78ab	3.08 ± 0.96a	2.47 ± 0.47ab	0.025
α-Terpinene	1-methyl-4-propan-2-ylcyclohexa-1,3-diene	Woody, terpenic, lemon	0.00 ± 0.00a	0.00 ± 0.00a	0.02 ± 0.00a	0.01 ± 0.00a	0.02 ± 0.02a	0.03 ± 0.01a	0.04 ± 0.01a	0.02 ± 0.02a	0.115
γ-Terpinene	1-methyl-4-propan-2-ylcyclohexa-1,4-diene	Oily, woody, terpenic	0.19 ± 0.01b	0.55 ± 0.45ab	1.22 ± 0.30ab	0.33 ± 0.45b	0.13 ± 0.16b	0.14 ± 0.12b	2.23 ± 0.92a	0.62 ± 0.35ab	0.013
α-Terpineol	2-(4-methylcyclohex-3-en-1-yl) propan-2-ol	Pine, terpenic, citrus	0.14 ± 0.01a	1.85 ± 0.10a	1.13 ± 0.15a	0.33 ± 0.04a	1.82 ± 0.73a	1.36 ± 1.93a	2.78 ± 1.00a	2.58 ± 0.08a	0.088
β-Citronellol	3,7-dimethyloct-6-en-1-ol	Citronella, rose, leafy	0.15 ± 0.21c	0.00 ± 0.00c	0.47 ± 0.17bc	1.79 ± 0.39ab	0.18 ± 0.13c	0.65 ± 0.92bc	0.89 ± 0.31abc	2.32 ± 0.33a	0.003
p-Cymene	1-methyl-4-propan-2-ylbenzene	Fresh, citrus, terpenic	0.01 ± 0.00a	0.05 ± 0.01a	0.03 ± 0.01a	0.02 ± 0.01a	0.10 ± 0.09a	0.05 ± 0.00a	0.06 ± 0.02a	0.03 ± 0.00a	0.357
Geraniol	(2E)-3,7-dimethylocta-2,6-dien-1-ol	Sweet, floral, fruity	1.03 ± 0.06ab	1.04 ± 0.16ab	1.28 ± 0.34ab	1.66 ± 0.00a	0.91 ± 0.35ab	0.18 ± 0.18b	1.64 ± 0.59a	1.36 ± 0.34ab	0.021
Hotrienol	(5E)-3,7-dimethylocta-1,5,7-trien-3-ol	Sweet, tropical, floral	0.01 ± 0.01a	0.01 ± 0.01a	0.02 ± 0.00a	0.01 ± 0.00a	0.04 ± 0.01a	0.02 ± 0.00a	0.04 ± 0.02a	0.68 ± 0.91a	0.476
Limonene	1-methyl-4-prop-1-en-2-ylcyclohexene	Citrus, orange, fresh	0.01 ± 0.00b	0.13 ± 0.06ab	0.12 ± 0.02ab	0.05 ± 0.02ab	0.16 ± 0.02ab	0.10 ± 0.10ab	0.23 ± 0.08a	0.15 ± 0.01ab	0.052

Linalool	3,7-dimethylocta-1,6-dien-3-ol	Citrus, floral, sweet	1.64 ± 0.07b	3.91 ± 0.62ab	8.17 ± 0.27ab	4.14 ± 0.04ab	5.81 ± 3.88ab	1.80 ± 2.54b	11.27 ± 4.21a	8.03 ± 1.27ab	0.027
Myrcene	7-methyl-3-methylideneocta-1,6-diene	Peppery, terpenic, spicy	0.37 ± 0.11b	1.16 ± 0.35ab	1.78 ± 0.14ab	0.08 ± 0.04b	1.45 ± 0.89ab	0.34 ± 0.40b	2.59 ± 1.07a	1.42 ± 0.00ab	0.018
Terpinolene	1-methyl-4-propan-2-ylidenecyclohexene	Fresh, woody, sweet	0.01 ± 0.00b	0.06 ± 0.00ab	0.04 ± 0.01ab	0.02 ± 0.01b	0.06 ± 0.01ab	0.06 ± 0.03ab	0.13 ± 0.05a	0.12 ± 0.01a	0.007
β-Damascenone	(E)-1-(2,6,6-trimethylcyclohexa-1,3-dien-1-yl) but-2-en-1-one	Natural, sweet, fruity	0.12 ± 0.01a	0.34 ± 0.00a	0.32 ± 0.01a	0.20 ± 0.01a	0.33 ± 0.01a	0.14 ± 0.19a	0.24 ± 0.06a	0.15 ± 0.05a	0.062
∑ TERPENES + NORISOPRENOIDS			*4.33*	*10.53*	*15.92*	*10.39*	*12.76*	*6.23*	*25.35*	*20.02*	
Sulfur-containing compounds											
Dimethyl sulfide	dimethyl sulfide	Cabbage, truffle	0.00 ± 0.00b	2.07 ± 1.32b	10.73 ± 0.53a	1.24 ± 0.01b	3.87 ± 3.75b	1.94 ± 0.19b	0.97 ± 0.18b	1.74 ± 0.91b	0.002
Methionol	3-methylsulfanyl propan-1-ol	Sulfurous, onion, sweet	0.00 ± 0.00c	8.50 ± 12.01bc	378.00 ± 31.66a	21.06 ± 0.10bc	77.86 ± 40.67b	26.62 ± 13.20bc	53.96 ± 4.45bc	19.49 ± 1.69bc	<0.0001
∑ SULFUR-CONTAINING COMPOUNDS			*0.00*	*10.56*	*388.73*	*22.30*	*81.72*	*28.56*	*54.93*	*21.22*	

Mean ± standard deviation values of two independent replicates are indicated. Different letters in each row indicate significant difference according to HSD Tukey test ($p<0.05$). OD = Odour Descriptor. <LOQ = below the limit of quantification.
